# Exploring manual asymmetries during grasping: a dynamic causal modeling approach

**DOI:** 10.3389/fpsyg.2015.00167

**Published:** 2015-02-24

**Authors:** Chiara Begliomini, Luisa Sartori, Diego Miotto, Roberto Stramare, Raffaella Motta, Umberto Castiello

**Affiliations:** ^1^Department of General Psychology and Center for Cognitive Neuroscience, University of PadovaPadova, Italy; ^2^Department of Medicine, University of PadovaPadova, Italy

**Keywords:** reach-to-grasp, hand dominance, functional magnetic resonance imaging, dynamic causal modeling

## Abstract

Recording of neural activity during grasping actions in macaques showed that grasp-related sensorimotor transformations are accomplished in a circuit constituted by the anterior part of the intraparietal sulcus (AIP), the ventral (F5) and the dorsal (F2) region of the premotor area. In humans, neuroimaging studies have revealed the existence of a similar circuit, involving the putative homolog of macaque areas AIP, F5, and F2. These studies have mainly considered grasping movements performed with the right dominant hand and only a few studies have measured brain activity associated with a movement performed with the left non-dominant hand. As a consequence of this gap, how the brain controls for grasping movement performed with the dominant and the non-dominant hand still represents an open question. A functional magnetic resonance imaging (fMRI) experiment has been conducted, and effective connectivity (dynamic causal modeling, DCM) was used to assess how connectivity among grasping-related areas is modulated by hand (i.e., left and right) during the execution of grasping movements toward a small object requiring precision grasping. Results underlined boosted inter-hemispheric couplings between dorsal premotor cortices during the execution of movements performed with the left rather than the right dominant hand. More specifically, they suggest that the dorsal premotor cortices may play a fundamental role in monitoring the configuration of fingers when grasping movements are performed by either the right and the left hand. This role becomes particularly evident when the hand less-skilled (i.e., the left hand) to perform such action is utilized. The results are discussed in light of recent theories put forward to explain how parieto-frontal connectivity is modulated by the execution of prehensile movements.

## INTRODUCTION

Human motor system organization is based on the principle of contralateral control of distal movement components, which is reflected at an anatomical level in a nearly complete cross-over of corticospinal fibers innervating distal muscles. It is known that the human brain is composed of two hemispheres that are not symmetrical, but specialized in some functions such as the motor control of the two hands. At the same time, right-hand dominance is considered evidence of a behavioral brain specialization, and 9 out of 10 individuals show a preference for right hand usage during most manual activities (Perelle and Ehrman, 1994). The question remains: how is right hand preference reflected in functional brain organization?

Recent neuroimaging techniques have made it possible to investigate the relationship between hand dominance and functional brain architecture. In this respect, functional magnetic resonance imaging (fMRI), electroencephalography (EEG), positron emission tomography (PET), magnetoencephalography (MEG), and transcranial magnetic stimulation (TMS) experiments have been recently utilized to study whether behavioral asymmetry (hand dominance) is associated with asymmetric neural tissue activation in the two hemispheres (Kim et al., 1993; Baraldi et al., 1999; Brouwer et al., 2001; Kobayashi et al., 2003; Pollok et al., 2006; Basso et al., 2006; Begliomini et al., 2008; Martin et al., 2011; Kourtis et al., 2014). Those studies have produced differing results in particular with regard to the activation of ipsilateral motor cortical areas in connection to the moving hand; the majority of fMRI studies has confirmed contralateral but also ipsilateral activation within motor-related areas (Kim et al., 1993; Baraldi et al., 1999; Kobayashi et al., 2003; Verstynen et al., 2005).

A point worth noting, however, is that it remains unclear whether activations are associated solely with higher order cortical areas and whether they regard only the non-dominant hand. Some studies report that hemispheric asymmetries in ipsilateral activations are present at the level of primary motor cortex (M1; Kawashima et al., 1993; Kim et al., 1993; Babiloni et al., 2003). Other studies seem to suggest that greater or lesser activation in the ipsilateral motor cortex is similar during left- or right-hand movements (Volkmann et al., 1998) and attribute hand dominance to a possible hemispheric asymmetry of higher order motor cortices such as premotor or supplementary motor areas (Hlustík et al., 2002). Despite the fact that the extent and magnitude of activation were found to be greater in the hemisphere contralateral to the hand being used (Culham and Valyear, 2006; Begliomini et al., 2008), recent fMRI evidence suggests that in right-handers grasping with either hand led to activation in the bilateral anterior intraparietal sulcus (AIP) and the right dorsal premotor cortex (dPMC; Begliomini et al., 2008). In this scenario, the control processes underlying hand dominance remain controversial for skilled movements. In part, this might be due to the measures used to identify unique attributes of the two hemispheres. Amongst these, the region of interest (ROI) method usually circumscribes the analysis to *a priori* defined brain regions within the left and the right hemispheres. As revealed by several studies, the precise localization of particular areas may vary across subjects (see Volkmann et al., 1998; Verstynen et al., 2005) and their anatomical size may differ across the left and right hemispheres (Amunts et al., 1996, 2000). The adoption of the ROI approach, thus, might represent a potential confound as it would run the risk of comparing regions that are functionally not quite equivalent in different individuals and different hemispheres.

With this in mind, here we considered the idea that the two hemispheres might contribute in different ways to the execution of grasping movements performed either with the left or the right hand. And to test this, we adopted the Dynamical Causal Modeling approach (DCM – Friston et al., 2003). DCM belongs to the family of effective connectivity approaches and has the potentiality of inferring about causality regulating functional couplings among brain regions. In our case, this peculiarity represents a potential key to disentangle a possible diverse contribution of the two hemispheres while performing grasping movements with the left or the right hand. We used DCM on fMRI time series (Friston et al., 2003) acquired during the execution of visually guided reaching-to-grasp movements toward a spherical object evoking precision grasping. This approach gives us the possibility to explore the inter-regional couplings between the main areas characterizing the grasping circuit in humans, that is the AIP together with the ventral premotor cortex (vPMC), the dPMC, and the M1 (Castiello, 2005; Castiello and Begliomini, 2008; Filimon, 2010).

Therefore the central aim of the present study was to verify whether, in right-handers, the execution of precision grip movements with either hand recruits the grasping circuit in a specular way [e.g., grasping with the right dominant hand (RDH) mainly recruits the left hemisphere and grasping with the left non-dominant hand (LNH) mainly recruits the right hemisphere] or whether hand dominance (i.e., RDH or LNH) could represent a crucial aspect for connectivity patterns among areas belonging to the grasping circuit. From this perspective, on the basis of available literature on both structural and functional data in both humans and monkeys (see **Table 1**), we hypothesized that the execution of precision grip movements with the LNH could modulate the connection between AIP areas of both hemispheres with respect to precision grip movements performed with the RDH. In fact, many studies have demonstrated bilateral AIP involvement when precision grip movements are performed with the dominant hand (Culham and Valyear, 2006; Davare et al., 2006, 2007). Since the left hand is less skilled, especially in performing precision movements (Gonzalez et al., 2006), we hypothesize that the execution of such movements with a not-skilled hand may require additional visuomotor processing, which could be provided by the contribution of both AIP areas. Alternatively, we hypothesized that, according to the model suggested by Rizzolatti and Luppino (2001), emphasizing the role of the connection AIP-vPMC in visuo-motor transformation underlying grasping movements, the connections between vPMCs could be ‘affected’ by precision grip movements performed with the LNH (see **Table 1**). Another plausible scenario could be represented by the possibility that the dPMC could be modulated by the execution of a precision grip movements performed with the LNH with respect to precision grip movements performed with the RDH, given the additional on-line control required by the execution of precision movements with the non-dominant hand (Begliomini et al., 2008). Finally, we also considered the hypothesis that the execution of a precision grip movement with the LNH does not modulate brain activity within the ipsilateral left hemisphere until execution. In this view, it might well be that it is the connection between the two primary motor areas to be modulated by the execution of a precision grip movement performed with the LNH.

**Table 1:** Studies supporting the existence of inter-hemispheric connections between grasping areas.

Connection	Non-human primate studies	Human primate studies
*AIP – AIP*		Tunik et al. (2005), Culham et al. (2006), Rice et al. (2006), Davare et al. (2007); Begliomini et al. (2008), Le et al. (2014)
*vPMC – vPMC*	Boussaoud (1995); Dancause et al. (2007)	
*dPMC – dPMC*	Marconi et al. (2003)	Begliomini et al. (2008)
*Ml – Ml*	Jenny (1979); Leichnetz (1986), Rouiller et al. (1994)	Davare et al. (2007)

*AIP, anterior intraparietal; vPMC, ventral premotor cortex; dPMC, dorsal premotor cortex; M1, primary motor cortex.*

**FIGURE 1:**
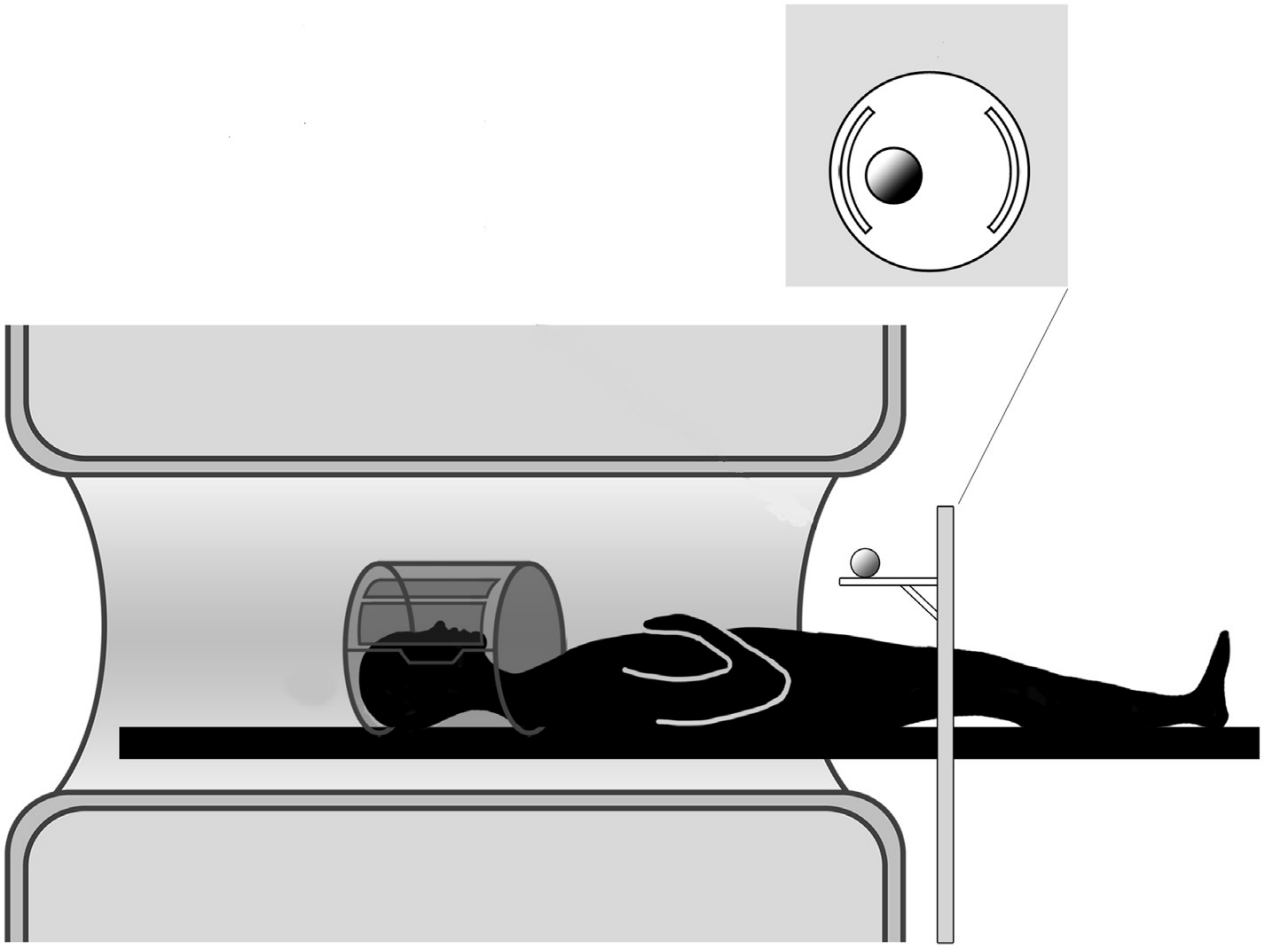
**Experimental setup.** The participant is lying in the MR scanner and the motorized platform ABRAM is presenting stimuli following a sequence administered by a PC located in the control room. The position of the rotating platform plus a pillow slightly tilting the head allow for direct viewing of the stimuli.

To summarize, the study focusses on the potential role played by hand dominance in the modulation of inter-hemispheric connections between homologs areas. In particular, on the basis of findings collected by previous studies from ours and other groups (Gonzalez et al., 2006, 2007; Begliomini et al., 2008 – see **Table 1**), we hypothesize that the execution of precision grip movements performed with the LNH could rely on the contribution of both hemispheres. Therefore, two possible main scenarios were considered (**Figure 1**):

(1) the execution of precision grip movements performed with the RDH modulates inter-hemispheric connections between homologs areas (models #1–4);
(2) the execution of precision grip movements performed with the LNH modulates inter-hemispheric connections between homologs areas (models #5–8);

The crucial point of the study is to examine which of the region/s belonging to the grasping circuit is/are involved by a hypothetical ‘encroachment’ to the ipsilateral hemisphere and therefore which aspect of grasping movement execution requires ‘additional’ resources to be provided by the ipsilateral hemisphere.

## MATERIALS AND METHODS

### PARTICIPANTS

Eighteen right-handed subjects (11 women and 7 men; age range: 19–30 years; mean age: 24.7 years) participated in the experiment. They all had normal or corrected-to-normal vision, and they had no neurologic or psychiatric history, or any motor pathology. Hand dominance was assessed by means of the Edinburgh Handedness Inventory (Oldfield, 1971). On the basis of the scores obtained with this test all participants were classified as strongly right-handed (36/36). Before entering the scanner room all participants underwent MR safety screening and gave informed written consent according to the guidelines provided by the Declaration of Helsinki. The study was approved by the local Ethics Committee.

### EXPERIMENTAL STIMULUS

The adopted stimulus consisted of a spherical plastic objects of 3 cm diameter presented at a constant distance of 30 cm. We used a regular geometric shape in order to make comparisons with macaque neurophysiology studies possible (Gallese et al., 1994; Umilta et al., 2007) and with the purpose to avoid confounds related to tool use, which is known to involve a particular network in the left-hemisphere (Johnson-Frey et al., 2005). The considered stimulus dimension was chosen to elicit a precision grip, which considers the opposition of thumb and index finger. The present investigation is confined to this kind of prehensile action since it has been well characterized in both neural (Ehrsson et al., 2001; Frey et al., 2005; Culham and Valyear, 2006; Begliomini et al., 2007a, 2014; Turella and Lingnau, 2014) and behavioral terms (e.g., Castiello et al., 1993; Jeannerod, 1981, 1984; Savelsbergh et al., 1996; Cuijpers et al., 2004; see Smeets and Brenner, 1999 for a review). Further, its accuracy requirements make it an ideal experimental framework to bold out the processes underlying planning and execution during grasping movements. With specific reference to neuroimaging studies, activation patterns registered during precision grip planning and execution appear to be characterized by a larger involvement of the parieto-frontal network with respect to other types of grasping movements (e.g., whole hand grasp – Begliomini et al., 2007a,b; see Filimon, 2010 for a review).

### EXPERIMENTAL SETUP

The stimulus was presented by means of an MR compatible motorized circular rotating table (ABRAM1; **Figure 1**). The participants’ upper arms were restrained with an elastic band to further minimize head movements consequent to arm movements. In order to keep the hand’s starting position constant across all participants and trials, the participants were asked to wear a metal-free belt cushioned by a pad and instructed to keep the performing hand (right or left) in a relaxed position with the palm placed face down on the pad. The other upper arm/hand unit was strapped to the scanner bore. Supported by a foam wedge, the participant’s head was tilted at an angle (∼30^∘^) to permit him/her to directly view the stimuli below the coil without needing mirrors; we were able, as a result, to avoid making other modifications that would have been required if mirror-viewing had been necessary (Culham et al., 2003; Cavina-Pratesi et al., 2007). While the participants were allowed to look freely between trials, they were explicitly instructed to look at the object throughout action execution.

### TASK PROCEDURES

The participants were requested to grasp the object, depending on the signal that was given, with either the RDH or the LNH hand using a precision grip. The participants were asked to grasp the object at a natural speed, depending on a sound (right hand: low tone – duration: 200 ms; frequency: 1,7 kHz; left hand: high tone – duration: 200 ms; frequency: 210 Hz.) delivered by means of pneumatic MR-compatible headphones wore by participants. Although the object was at all times visible, the participants was instructed to begin the movement only upon hearing the sound. An operator in the control cabin next to the scanner room monitored the entire experiment. In particular, she checked that the participants fulfilled the task requirements in terms of grasping actions.

### EXPERIMENTAL DESIGN

The experiment was conducted by using a mixed event-related design. The performing hand (RDH, LNH) was manipulated within runs as within-subjects factor. Trials to be performed with the same hand were grouped in sequences varying from four to eight elements. This was done in order to minimize brain activity due to frequent task changes (Culham et al., 2003). In accordance with a ‘long exponential’ probability distribution, the inter-stimulus interval (ISI), which was randomized across trials, varied from 3 to 8 s (Hagberg et al., 2001). An entire experimental session consisted of 120 trials, which were divided into two runs (kept short to minimize participants’ fatigue) of 60 trials each per condition.

### IMAGING PARAMETERS

Images were acquired by means of a whole-body 1.5 Tesla scanner (Siemens Magnetom Avanto) equipped with a standard Siemens coil (eight channels). Functional images were acquired with a gradient-echo, echo-planar (EPI) T2^∗^-weighted sequence in order to detect blood oxygenation level-dependent (BOLD) contrast throughout the whole brain (37 axial slices acquired continuously with descending order, 56 × 64 voxels, 3 mm × 3 mm × 3.3 mm resolution, FOV = 196 mm × 224 mm, flip angle = 90^∘^, TE = 49 ms). 114 volumes were collected continuously in each single scanning run (TR: 3 s), resulting in two functional runs of 5 m and 42 s duration (11 m and 24 s of acquisition time in all). High-resolution T1-weighted anatomical image was acquired for each participant (3DMP-RAGE, 176 axial slices, no interslice gap, data matrix 256 × 256, 1 mm isotropic voxel, TR = 1900 ms, TE = 2.91 ms, flip angle = 15^∘^).

### DATA ANALYSIS

#### Data preprocessing

Functional data were spatially pre-processed and analyzed with SPM8 (Statistical Parametric Mapping^1^). The first four scans for each session were discarded from data analysis to avoid effects due to the non-equilibrium state of magnetization. For each participant, the time series for each voxel was realigned temporally to acquisition of the middle slice and underwent motion correction, realigning each volume to the first in the series. The anatomical scan was then co-registered to the mean of all functional images, previously corrected for intensity inhomogeneities through the bias correction algorithm implemented in SPM8. EPI images were then normalized according to the MNI152 template, supplied by the Montreal Neurological Institute^2^ and distributed with the software SPM8. Finally, images were smoothed using a 6 mm× 6 mm× 6.6 mm FWHM 3D Gaussian kernel (twice the native voxel size). After motion correction two participants had to be excluded from further analysis because of large head motion (exceeding voxel size, 4 mm).

#### General linear model

At the first level, for each single participant, movements performed either with the RDH or the LNH were modeled as separate regressors with a General Linear Model (GLM - Friston et al., 1995). The duration of the movement was assumed of about 1.5 s on the basis of behavioral observations before the experimental session, done in order to get participants acquainted with the experimental setup. Regressors were defined on the timing of presentation of each experimental condition (cueing sound). These functions were convolved with a canonical, synthetic haemodynamic response function (HRF) plus temporal derivative to produce individual models (Henson et al., 2001). For each subject, both regressors were incorporated into General Linear Models (Holmes et al., 1997). Further, motion correction parameters, created during the realignment stage, missed trials, errors as well as the remaining part of the movement (the hand going back from the object to the starting position) were included in the analysis as a covariate of no interest. This was done in order to model residual effects due to head motion and factors of no interest. Individual models were separately estimated and contrasts were defined in order to pick out the main effects of each experimental condition. Time series data were concatenated over the sessions, and two regressors of no interest were added to the model to account for session effects.

#### DCM models

**FIGURE 2:**
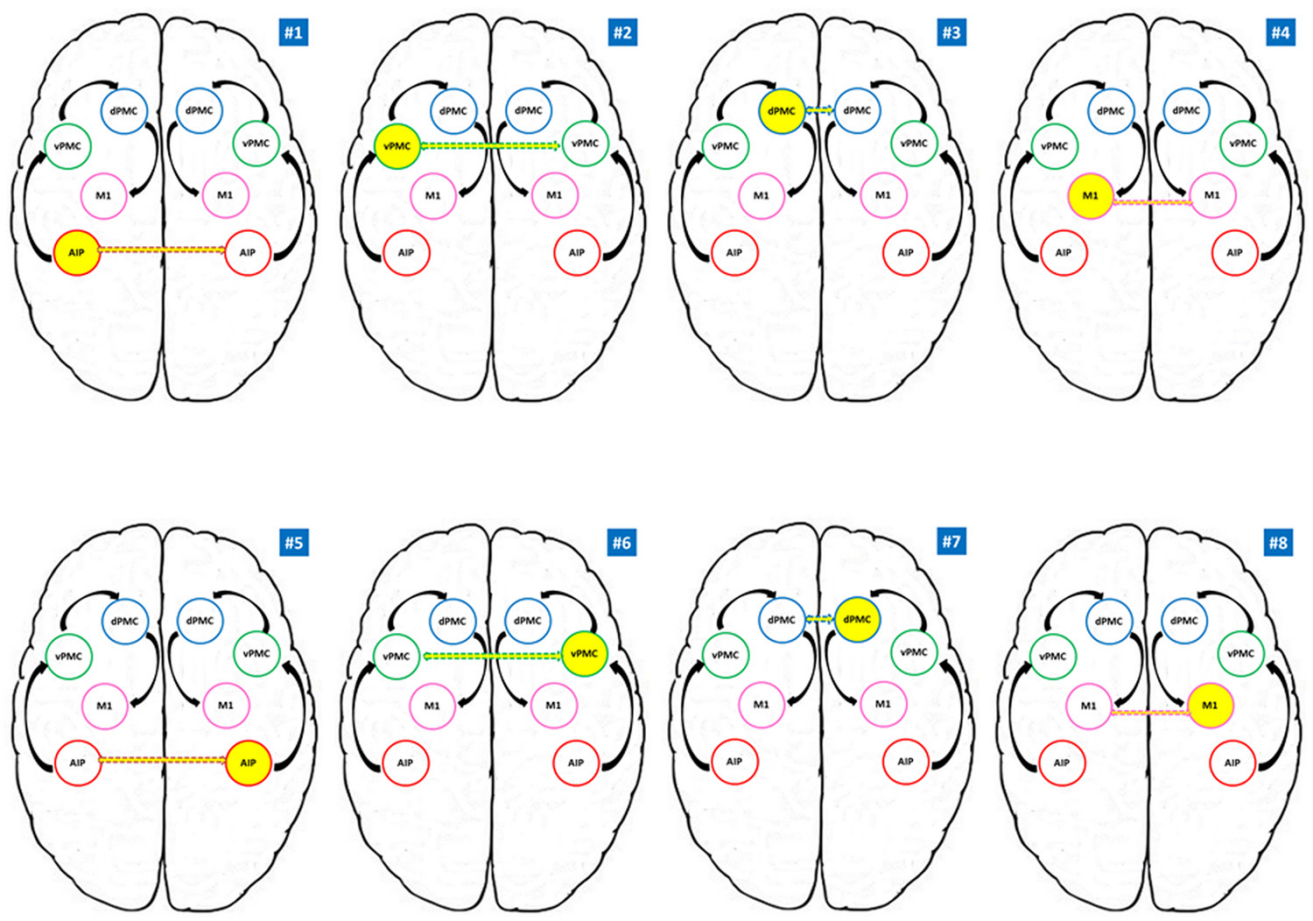
**Models tested for the RFX Bayesian Model Selection (BMS).** AIP, anterior intraparietal; vPMC, ventral premotor cortex; dPMC, dorsal premotor cortex; M1, primary motor cortex. Models #1 to #4 belong to the RDH family; models #5 to #8 refer to the LNH family. Yellow circles indicate the modulating region while dotted arrows indicate the connection to the homologous region in the other hemisphere. Black arrows indicate the intra-hemispheric structure of the model.

The question that the DCM tries to address in this study is concerned with the hypothesis that precision grip movements performed with the RDH or the LNH could modulate inter-hemispheric connections between homologous areas (e.g., right AIP–left AIP) in different ways, according to the models described in **Figure 2**.

We hypothesized intra- and inter-hemispheric connections among the grasping key regions (AIP, vPMC, dPMC, and M1) on the basis of results obtained by single cell recordings performed on macaque monkeys (see **Table 1**) and referring to the model described by Castiello and Begliomini (2008). More in detail, whereas for inter-hemispheric connections between dPMC, vPMC, and M1 we can rely on neurophysiological data, concerning AIP we mainly refer to the results obtained in humans by means of neuroimaging techniques such as fMRI (Culham et al., 2006; Begliomini et al., 2008) and TMS – (Tunik et al., 2005; Rice et al., 2006; Le et al., 2014). Overall these studies seem to converge on the hypothesis of a bilateral contribution of AIP to grasping execution.

For each participant eight different models, considering eight different connectivity hypothesis were tested (see **Figure 2**). We considered anatomical models consisting of volumes of interest (VOIs) with reciprocal connections between them (DCM-A matrix) according to the considered theoretical model (Castiello and Begliomini, 2008). The visuomotor analysis of the to be grasped object served as driving input (matrix C), and therefore we considered AIP as the driving input area in each hemisphere, given its crucial role in such processes (Binkofski et al., 1998, 1999; Frey et al., 2005; Rice et al., 2006, 2007; Begliomini et al., 2007a). In our models, we did exclude any hypothesis related to stimulus-response coupling dynamics (sound → performing hand) since the present work focuses on grasping execution rather than planning.

According to our reference model (Castiello and Begliomini, 2008), the modulation induced by our experimental task is supposed to propagate through connections from AIP to vPMC, and from vPMC to dPMC. The subsequent connection is supposed to link dPMC with ipsilateral M1, which is assumed to be the final node of our models (see **Figure 2**). The performing hand (RDH; LNH – DCM-B matrix) served as a modulatory influence on the forward connections. We adopted the models #1–4 as ‘RDH’ family model since they do hypothesize inter-hemispheric interaction between homologous areas as driven by precision grip movements performed with the RDH (model #1: left AIP ↔ right AIP; model #2: left vPMC ↔ right vPMC; model #3: left dPMC ↔ right dPMC; model #4 left M1 ↔ right M1). Similarly, models #5, #6, #7, and #8 hypothesize the same structure, where the inter-hemispherical connection between homologous areas is modulated by precision grip movements performed with the LNH (‘LNH’ family; model #5: right AIP ↔ left AIP; model #6: right vPMC ↔ left vPMC; model #7: right dPMC ↔ left dPMC; model #8 right M1 ↔ left M1).

#### VOI definition

The relevant time series of the regions included in the DCM analysis were extracted from the fMRI data of each individual subject on the basis of event-related analyses in the context of the General Linear Model. The VOIs were both functionally and anatomically located: (i) for each participant, the *t-contrast* testing for the global effect of the experimental manipulation (precision grip movements performed with RDH + precision grip movements performed with LNH) was considered (*p* < 0.001, uncorrected for multiple comparisons); (ii) this contrast was inclusively masked by the image resulting from the overlap *between* activation maps detected for each precision grip movement. This procedure was chosen in order to detect brain regions commonly involved by both movement without applying any statistical threshold; (iii) The small volume correction (Worsley et al., 1996) was performed on the resulting masked activation image by adopting the cytoarchitectonic maps provided by the toolbox Anatomy (Eickhoff et al., 2007) as searching areas. The following maps were selected: anterior intraparietal sulcus (Choi et al., 2006; Scheperjans et al., 2008), Broca’s region (Amunts et al., 1999), the motor cortex (Geyer et al., 1996), and the premotor cortex (Geyer, 2003). The first set of coordinates detected for each area (AIP left, AIP right, vPMC left, vPMC right, dPMC left, dPMC right, M1 left, and M1 right) was chosen as the reference for the creation of the VOI. More in detail for M1 VOIs the chosen coordinate had to be located in the precentral gyrus, near the ‘hand knob’ (Yousry et al., 1997) while for the dPMC coordinates provided by Davare et al. (2006) were taken as a reference point to define the dorsal region of the premotor cortex. For each participant, a spherical VOI of 5 mm radius was built around the first set of coordinates detected with the SVC procedure in each of all the eight regions included in the analysis. The time series for each VOI was extracted by considering the ‘effects of interest’ (*t-contrast*) and adjusted for the ‘effects of no interest’ (*F-contrast*), including regressors of no interest (motion parameters, errors, missed trials, and time intervals needed by the hand to go back to the starting position after the movement). The percentage of variance observed for each regions was above 75% in all cases.

#### Model estimation and selection

In order to verify our hypothesis concerning laterality of the involvement of grasping areas during precision grip movements performed with the LNH and the RDH, we applied Bayesian inference to the hypothesized models (Penny et al., 2004). Bayes factors (i.e., ratios of model evidences) were used to compare different models. The estimated models were compared, based on the model evidences *p* (*y*|*m*), which is the probability *p* of obtaining observed data *y* given by a particular model *m* (Friston et al., 2003; Stephan et al., 2009). Bayesian model selection (BMS) was performed with a random effects analysis using a Gibbs sampling method (Stephan et al., 2009; Penny et al., 2010). This method accounts for the possibility that different models apply to different subjects. Model comparison was (i) first done at the level of model families, i.e., subsets of models that share particular attributes. Two different model families were created, defined on the basis of the modulation hypothesis of connections (RDH-driven or LNH-driven). After that, (ii) we focused on the winning family considering the most significant modulation effect induced by our task.

The selection of a model yields the exceedance probability for each model family/model, which express the probability (in %) that a particular family/model is more likely than any other. Exceedance probabilities for all families/models sum to 100%.

## RESULTS

### GLM GROUP ANALYSIS RESULTS

Prior to conducting the DCM analyses described above, a conventional second-level Random Effect Analysis (RFX) was conducted on the HRF for the whole brain volume (*p* < 0.005, *FDR-corrected* for multiple comparisons, *k* > 12) as to confirm the involvement of motor, premotor, and parietal regions in our task. The contrast of interest tested for specific effects of precision grip movements performed with the RDH or with the LNH. These contrasts identified activation of cortical areas consisting of primary motor and premotor cortices, as well as parietal areas (see **Table 2**). In particular, while activity associated with precision grip movements performed with the RDH appeared to be more circumscribed to the left contralateral hemisphere, activity observed for precision grip movements performed with the LNH involved dorsal premotor and parietal regions of both hemispheres. The group analysis did not reveal any significant activity in the left vPMC, which was observed by means of a small volume correction (Worsley et al., 1996) instead. As described in the ‘VOI definition’ section, the VOIs were located for each participant following both functional and anatomical criteria. This procedure ensured that the functional regions included in the DCM models were as consistent as possible across subjects (Stephan et al., 2007; Seghier et al., 2011). Coordinates for each single region in each participant are reported in Table 1 of the Supplementary Material. No significant effects were observed for the same analysis procedure conducted on the time derivative included in the GLM model.

**Table 2:** Results of the RFX analysis performed on the whole group (*p* < 0.005, FDR-corrected for multiple comparisons, *k* > 12).

Cluster level			Peak level				MNI					
*p(FWE)*	*k*	*p(unc)*	*p(FDR)*	*t*	*Z-score*	*p(unc)*	*X*	*Y*	*Z*	*Side*	*Region*	*BA*
**0.000**	**1339**	**0.000**	**0.000**	**10.711**	**6.821**	**0.000**	**-48**	**-69**	**7**	**L**	**MTG**	**39**
			0.000	8.567	6.047	0.000	-55	-56	16	L	STG	22
			0.000	6.903	5.300	0.000	14	-72	22	R	PRECU	31
**0.000**	**347**	**0.000**	**0.000**	**10.478**	**6.746**	**0.000**	-**35**	-**20**	**64**	**L**	**PRECG**	**4**
			0.000	8.716	6.107	0.000	-38	-13	58	L	PRECG	6
			0.000	7.760	5.704	0.000	-42	-39	58	L	IPL	40
**0.000**	**651**	**0.000**	**0.000**	**9.415**	**6.375**	**0.000**	**41**	-**20**	**49**	**R**	**PRECG**	**4**
			0.000	8.051	5.832	0.000	47	-13	52	R	PRECG	4
			0.000	7.873	5.754	0.000	41	**-**13	58	R	PRECG	6
**0.030**	**32**	**0.008**	**0.000**	**7.033**	**5.364**	**0.000**	**11**	**7**	-**11**	**R**	**PUTAMEN**	
			0.002	4.435	3.858	0.000	21	13	-11	R	PUTAMEN	
**0.016**	**39**	**0.004**	**0.000**	**6.633**	**5.164**	**0.000**	**28**	-**56**	**55**	**R**	**SPL**	**7**
**0.000**	**125**	**0.000**	**0.000**	**6.469**	**5.079**	**0.000**	**54**	-**66**	**1**	**R**	**MTG**	**37**
			0.001	5.248	4.386	0.000	54	-63	19	R	STG	39
**0.034**	**31**	**0.009**	**0.000**	**5.818**	**4.723**	**0.000**	**21**	-**79**	**46**	**R**	**PRECU**	**7**
**0.107**	**20**	**0.029**	**0.002**	**4.513**	**3.911**	**0.000**	-**42**	-**30**	**31**	**L**	**POCG**	**2**
**0.239**	**13**	**0.071**	**0.001**	**5.039**	**4.256**	**0.000**	**51**	**0**	**25**	**R**	**IFG**	**9**
**0.037**	**30**	**0.010**	**0.001**	**5.008**	**4.236**	**0.000**	**44**	-**3**	**7**	**R**	**INSULA**	**13**
			0.005	3.847	3.441	0.000	51	10	10	R	IFG	44
**0.107**	**20**	**0.029**	**0.001**	**4.964**	**4.208**	**0.000**	**21**	-**6**	**10**	**R**	**GL. PALLIDUS**	
**0.239**	**13**	**0.071**	**0.002**	**4.481**	**3.890**	**0.000**	**8**	**-59**	**-35**	**R**	**CEREBELLUM**	
**0.304**	**15**	**0.093**	**0.002**	**4.386**	**3.825**	**0.000**	**21**	**-46**	**-47**	**R**	**CEREBELLUM**	
**0.079**	**12**	**0.451**	**0.090**	**3.791**	**3.399**	**0.000**	**-48**	**17**	**-2**	**L**	IFG*	**45**
			0.090	3.425	3.121	0.001	**-**52	20	**-**5	L	IFG*	45

The considered contrast is precision grip_RDH + precision grip_LNH. MTG, middle temporal gyrus; STG, superior temporal gyrus; PRECU, precuneus; PRECG, precentral gyrus; IPL, inferior parietal lobule; SPL, superior parietal lobule; POCG, post central gyrus; IFG, inferior frontal gyrus. Bolded font indicates the first activation peak of the cluster (in terms of *t* and *Z* score). *results obtained by meansof a small volume correction.

### DCM RESULTS

Effective connectivity was tested by DCM-10, implemented in SPM8 toolbox (Wellcome Department of Imaging Neuroscience, London, UK), running under Matlab R2011a (The MathWorks, Natick, MA, USA).

#### Family wise results

**FIGURE 3:**
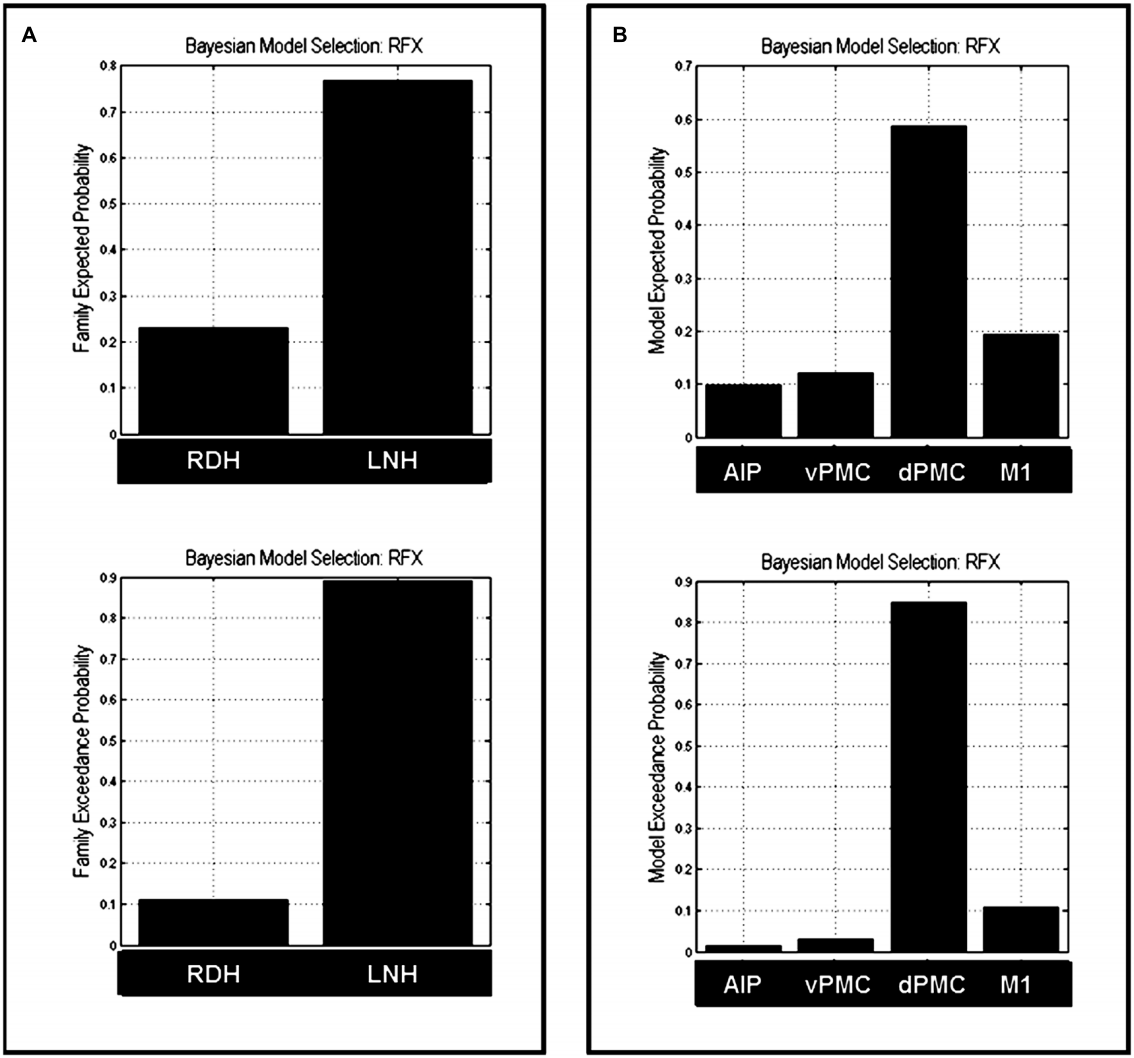
**Results of the BMS RFX performed at the family level **(A)** and at the model level **(B)**.** For both levels, expected (upper panels) and exceedance probabilities (lower panels) are reported. RDH, right dominant hand; LNH, left non-dominant hand; AIP, anterior intraparietal; vPMC, ventral premotor cortex; dPMC, dorsal premotor cortex; M1, primary motor cortex.

Bayesian Model Selection was used first to decide which family model (RDH or LNH) better explains the measured data. The results showed that the ‘LNH’ family had an exceedance probability of 0.8902 compared to the ‘RDH’ family (0.1098; see **Figure 3A**). The winner family contains four models hypothesizing inter-hemispheric connections between homologs areas (AIP, vPMC, dPMC, and M1) as ‘influenced’ by precision grip movements performed with the LNH, which assumes that the modulation of connections starts from the right hemisphere.

#### Model-wise results

As a second step, we performed a RFX analysis on the four models belonging to the ‘LNH’ family and, as reported in **Figure 3B**, the ‘dPMC’ model is associated with the highest exceedance probability (0.847), followed by the ‘M1’ model (0.108) and the ‘vPMC’ model (0.029). The probability value associated with the ‘AIP’ model was even below 5% (0.014). This result indicates that, among the models we considered in the study, the ‘winner’ is characterized by bidirectional connections between dPMC areas of the two hemispheres.

**FIGURE 4:**
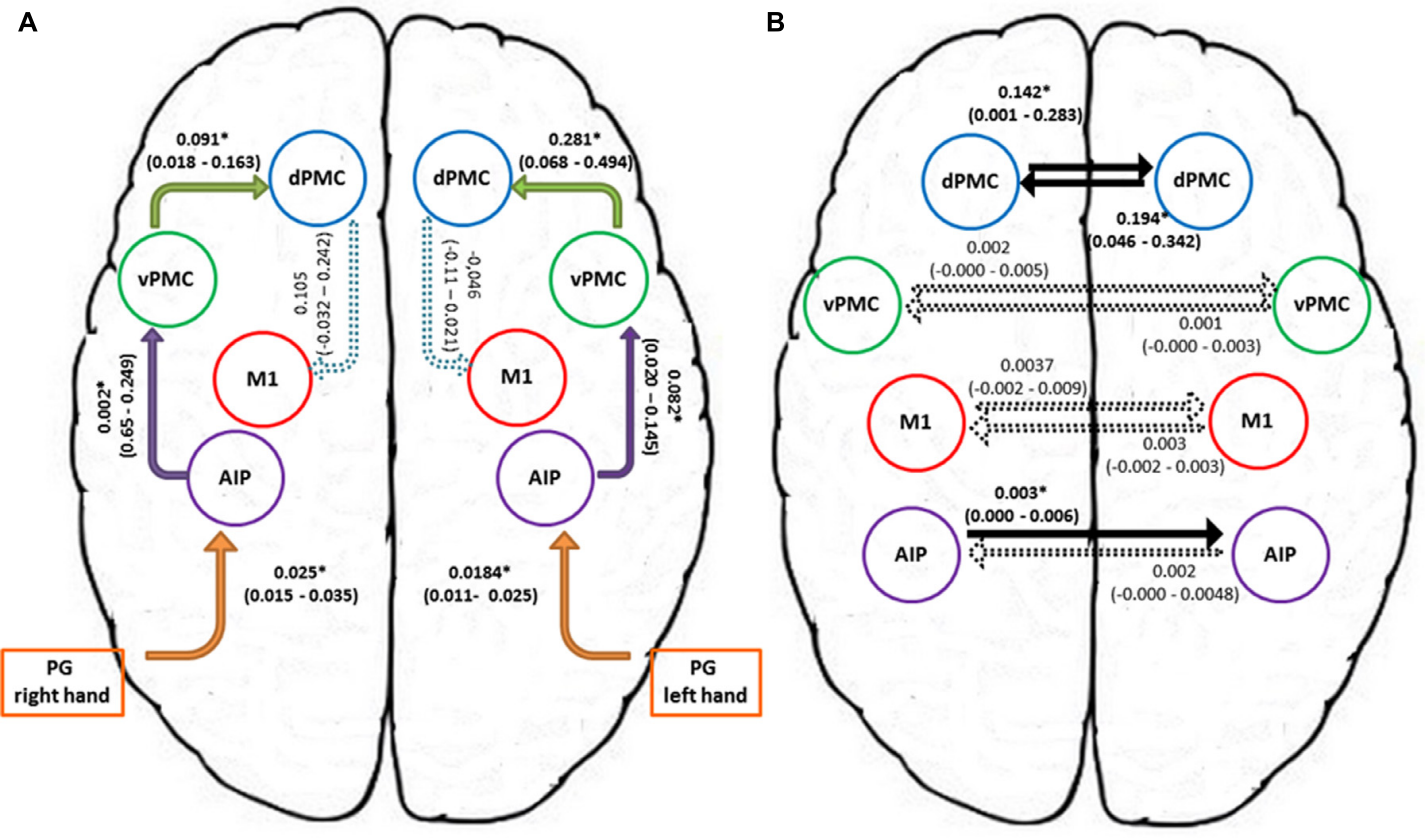
**Connection strengths of the tested models. (A)** Shows intra-hemispheric connections and **(B)** shows inter-hemispheric connections. Solid lines indicate significant modulation effects. Group-level averages of MAP estimates and 95% confidence intervals are illustrated. The averages were tested against 0 and significant results are signified with ∗ if *p* < 0.05. AIP, anterior intraparietal; vPMC, ventral premotor cortex; dPMC, dorsal premotor cortex; M1, primary motor cortex; PG, precision grip.

In order to further characterize the peculiarities of the modulation induced on the connections of the winner model, parameter estimates resulting from Bayesian Model Averaging (BMA) were extracted for each connection of the models belonging to the winning family and were tested against 0 (one-sample *t*-test, *p* < 0.05) to verify whether a significant modulation was present. The results are reported in **Table 3** and depicted in **Figure 4A**. The statistical analysis revealed that grasping with both hands significantly modulated the selected input regions (namely AIP left for precision grip movements performed with RDH *t*_(15)_ = 5.465 *p* < 0.000, and AIP right for precision grip movements performed with the LNH, *t*_(15)_ = 5.788 *p* < 0.000). Concerning the left hemisphere, which is supposed to be primarily involved in the control of precision grip movements performed with the RDH (**Figure 4A**) the connections AIP-vPMC and vPMC-dPMC appeared as significantly modulated [namely *t*_(15)_ = 3.649 *p* = 0.002; *t*_(15)_ = 2.686 *p* = 0.017]. The connection between dPMC and M1 did not show any significant modulation effect. Concerning the right hemisphere, which is supposed to be primarily involved in the control of precision grip movements performed with the LNH (**Figure 4A**), the connections AIP-vPMC as well as vPMC-dPMC are significantly modulated, similarly to the left hemisphere [*t*_(15)_ = 2.815, *p* = 0.013; *t*_(15)_ = 2.820, *p* = 0.013]. Also for the right hemisphere, the dPMC-M1 connection did not appear as significantly modulated. When looking at inter-hemispheric connections between homologous areas (**Table 4A**; **Figure 4b**), the connection between AIPs appears to be significantly modulated in the L → R direction but not viceversa [*t*_(15)_ = 2.563, *p* = 0.022 vs. *t*_(15)_ = 1.705 *p* = 0.109]. Concerning dPMC, the connection appears to be modulated in both directions (L → R *t*_(15)_ = 2.158, *p* = 0.048; R → L *t*_(15)_ = 2.801, *p* = 0.013]. No further significant results were observed concerning analysis performed on individual connections.

**Table 3:** Results obtained by one-sample *t*-tests performed on the parameter estimates related to input effects, inter-regional, and modulatory connections of the winning family LNH (*p* < 0.05).

	INPUT	AIP LEFT	AIP RIGHT	vPMC LEFT	vPMC RIGHT	dPMC LEFT	dPMC RIGHT	M1 LEFT	M1 RIGHT
**AIP LEFT**	*t*_(15)_ = 5.465***p* < 0.000**		*t*_(15)_ = 1.705 *p* = 0.109						
**AIP RIGHT**	*t*_(15)_ = 5.788***p* < 0.000**	*t*_(15)_ = 2.563***p* = 0.022**							
**vPMC LEFT**		*t*_(15)_ = 3.649***p* = 0.002**			*t*_(15)_ = 1.929 *p* = 0.073				
**vPMC RIGHT**			*t*_(15)_ = 2.815***p* = 0.013**	*t*_(15)_ = 1.946 0.071					
**dPMC LEFT**				*t*_(15)_ = 2.686***p* = 0.017**			*t*_(15)_ = 2.801***p* = 0.013**		
**dPMC RIGHT**					*t*_(15)_ = 2.820***p* = 0.013**	*t*_(15)_ = 2.158***p* = 0.048**			
**M1 LEFT**						*t*_(15)_ = 1.632 *p* = 0.123			*t*_(15)_ = 0.245 *p* = 0.809
**M1 RIGHT**							*t*_(15)_ = -1.471 *p* = 0.162	*t*_(15)_ = 1.321 *p* = 0.206	

*AIP, anterior intraparietal; vPMC, ventral premotor cortex; dPMC, dorsal premotor cortex; M1, primary motor cortex. Table has to be read as follows: cells on top of the columns are the ‘input’ region and rows represent the ‘target.’ Bold values in the table indicate significant results.*

**Table 4A:** Results obtained by paired *t*-test performed on the parameter estimates related to LEFT → RIGHT connections strengths of the winning family LNH (*p* < 0.05).

	AIP_LEFT AIP_RIGHT (0.0035)	vPMC_LEFT vPMC_RIGHT (0.0026)	dPMC_LEFT dPMC_RIGHT (0.142)	M1_LEFT M1_RIGHT (0.0037)
**AIP_LEFT** AIP_RIGHT **(0.0035)**		*t*(15) = -0.695 *p* = 0.498	*t(15)* = -*2.119 p* = 0*.051*	*t*(15) = -0.067 *p* = 0.948
**vPMC_LEFT** vPMC_RIGHT (0.0026)			*t(15)* = -*2.116 p = 0.051*	*t*(15) = -0.067 *p* = 0.948
dPMC_LEFT dPMC_RIGHT (0.142)				*t*(15) = -0.067 *p* = 0.948
M1_LEFT M1_RIGHT (0.0037)				

*AIP, anterior intraparietal; vPMC, ventral premotor cortex; dPMC, dorsal premotor cortex; M1, primary motor cortex. Numbers in title column/row indicate the parameter estimate obtained for that connection.*

**Table 4B:** Results obtained by paired *t*-test performed on the parameter estimates related to RIGHT LEFT connections strenghts of the winning family LNH (*p* 0.05).

	AIP_RIGHT AIP_LEFT (0.0021)	vPMC_RIGHT vPMC_LEFT (0.0018)	dPMC_RIGHT dPMC_LEFT (0.194)	dPMC_RIGHT dPMC_LEFT (0.194)
**AIP_RIGHT AIP_LEFT (0.0021)**		*t*(15) 0.236 *p* 0.816	***t*(15) 2.758 *p* 0.015**	*t*(15) 1.477 *p* 0.160
**vPMC_RIGHT vPMC_LEFT (0.0018)**			***t*(15) 2.765 *p* 0.014**	*t*(15) 1.202 *p* = 0.248
**dPMC_RIGHT dPMC_LEFT (0.194)**				***t*(15) 2.804 *p* 0.013**
**M1_RIGHT M1_LEFT (0.0003)**				

*AIP, anterior intraparietal; vPMC, ventral premotor cortex; dPMC, dorsal premotor cortex; M1, primary motor cortex. Bold values in the table indicate significant results.*

More in detail, paired *t*-tests were also conducted to test for differences between inter-hemispheric connections, in order to examine more in depth the results highlighted by the BMA. The results (**Tables 4A,B**) show that connections from the left toward the right hemisphere do not differ in terms of strength. It is worth mentioning that the modulation exhibited from the dPMC_LEFT toward the dPMC_RIGHT almost reaches significance with respect to all the other considered LEFT → RIGHT connections [dPMC-AIP: *t*_(15)_ = -2.119, *p* = 0.051; dPMC-vPMC: *t*_(15)_ = -2.116,.051; *t*_(15)_ = 2.089, *p* = 0.054]. Differently, when looking at RIGHT → LEFT connections, the modulation effect exhibited by the connection between dPMC areas significantly differs from the others (dPMC-AIP: *t*_(15)_ = -2.758, *p* = 0.015; dPMC-M1: *t*_(15)_ = -2.765, *p* = 0.014; *t*_(15)_ = -2.804, *p* = 0.013]. No further significant effects were observed.

## DISCUSSION

We used DCM to evaluate whether and how the intra- and inter-hemispheric couplings between brain areas composing the parieto-frontal network underlying grasping movements were modulated by the used hand. To test this hypothesis, right-handed participants were requested to perform reach to grasp movements toward and grasp an object with either the right or the left hand. The relative simplicity of the motor task enabled us to obtain robust coupling parameters between key areas of the grasping circuit.

In general, we showed that when right-handers perform a precision grip movement with the RDH it is the left hemisphere to be chiefly involved. However, when they perform a precision grip movement with the LNH the ipsilateral hemisphere is also involved. More specifically, such involvement appears to be confined at the level of the dPMC and to a lesser extent at the level of the AIP and the vPMC.

Some functional imaging studies in which neurovascular responses that were evoked during visually guided grasping movements by right-handers were localized, demonstrated that there was increased activity in the region situated between the intraparietal and the inferior postcentral sulci (AIP; ; Toni et al., 2001; Culham et al., 2003; Begliomini et al., 2007a, 2008) and in the ventral portion of the precentral gyrus (vPMC; Toni et al., 2001). Similar activities were also noted during object manipulation studies (Binkofski et al., 1999; Ehrsson et al., 2000; Johnson-Frey et al., 2005).

In terms of effective connectivity, previous results (Grol et al., 2007) showed that there are specific, differential changes in effective connectivity between AIP and VPM during reaching-to-grasp movements. A finding that fits with the general notion that the dorsolateral circuit is concerned with controlling grasping parameters of the prehension movement (Jeannerod et al., 1995). Along these lines, the present study shows that when precision grip movements are performed with the right hand, the connections “AIP-vPMC” and “vPMC-dPMC” within the left hemisphere appeared to be significantly modulated. In a similar vein, the “AIP-vPMC” as well as the “vPMC-dPMC” connections were modulated within the right hemisphere, which is supposed to be primarily involved in the control of precision grip movements performed with the left non-dominant hand.

The revelation of “vPMC-dPMC” connections is particularly important because it confirms a series of neurophysiological studies demonstrating an intra-hemispheric cross-talk between these two areas. An important aspect of the neurons recorded in the dPMC area F2 in macaques, is that they showed very similar properties to those previously described in the vPMC area F5 (Murata et al., 1997; Rizzolatti and Fadiga, 1998). Therefore, it has been advanced that both areas F2 and F5 may collaborate in the control of grasping actions. In this respect, Raos et al. (2004) pose an interesting question. That is, why are two premotor areas involved in grasping actions? In this respect, these authors posited that area F5 is chiefly concerned with the selection of the most appropriate type of grip (Raos et al., 2004). This motor representation is then supplied to area F2 whose neurons presumably keep a memory trace of the selected motor representation as to continuously update hand configuration and orientation while it approaches the object to be grasped.

When looking at inter-hemispheric connections between homologous areas the connection between the right and the left AIPs appears to be significantly modulated for the ‘left to right’ direction but not viceversa. In both humans and monkeys AIP is a crucial component of the parietal-premotor circuit known to be involved in the ‘translation’ of object intrinsic properties into specific grips (Rizzolatti and Luppino, 2001). In the present study, we confirm the pattern of a bilateral involvement of AIP, previously found in right-handers using either the right or the left hand (Davare et al., 2007).

However, we further deepen these findings suggesting that there is no bidirectional crosstalk between the two homologous areas, or that such cross-talk could be rather limited to the ‘left-right’ direction. Indeed, hand shaping during TMS studies appeared to be impaired only when TMS was applied bilaterally to AIP (Davare et al., 2007), while when the AIP virtual lesion was unilateral hand shaping remained intact. The existence of a cross-talk would seem to explain this finding, and both AIPs seemed necessary regardless of the hand being use (Davare et al., 2007). Two further studies demonstrated that unilateral AIP lesions are unable to alter the ability to shape the hand as to grasp the object hand conformation except when object size and orientation are modified unexpectedly (Tunik et al., 2005; Rice et al., 2006).

As these findings concern grasping execution, they support the hypothesis that a bilateral AIP involvement is required for precision grip movements and that this aspect is a distinctive feature of the anterior sector of the posterior parietal cortex (for review see Castiello, 2005; Culham et al., 2006; Castiello and Begliomini, 2008; Filimon, 2010). Noticeably, in the present study the pattern of connectivity found within this area has a specific direction depending on the hand used. In particular, an increase in connectivity appears to be evident when right-handers use the left hand and, therefore, the right hemisphere is chiefly involved. In fact, inter-hemispheric connections between homologous areas appear to be boosted mainly for the right-left direction when the LNH is used, as if the accomplishment of a precision grip movement with the LNH would require additional processing coming from the left, dominant hemisphere. The superiority of the right hand in high precision inter-joint coordination and in performing dexterous finger movements and trajectory formation has been observed in right-handers (Healey et al., 1986). The accuracy required by the task described in the study presented here and the evident need to determine precise contact points both point to right hand superiority in right-handers, suggesting that when the precision grip movement is performed by the RDH, the left AIP is able to accomplish the sophisticated visuomotor transformation underlying this movement without ‘contributions’ coming from its homologous in the right hemisphere.

In contrast to the AIP, the connection amongst the right and left dPMC appears to be modulated in both directions. More specifically, as outlined by the BMA results, the modulation of the connections from the left to the right dPMC almost reached significance. In contrast the remaining ‘left to right’ connections were far from being significant (see **Table 4A**). When looking at the ‘right–left’ (**Table 4B**) connections, the modulation effect exhibited by the connections between the dPMC appears to be stronger in comparison with all the other inter-hemispheric connections, suggesting that the modulation effect induced by a precision grip movement performed with the LNH is maximally expressed in terms of on-line monitoring ‘contribution,’ accomplished by the dPMC (Davare et al., 2006; Begliomini et al., 2008).

To summarize, when comparing the strength of interhemispheric connections it is evident that for the ‘left to right’ direction there are no differences. However, when comparing ‘right to left’ interhemispheric connections, the connection between the right and left dPMC is much stronger than the connection between the AIP, vPMC, and M1 and their homologous in the left hemisphere. This might indicate that when the precision grip movements is performed with the LNH the ipsilateral dPMC is recruited to a higher extent. In other words, the right hemisphere is in charge of the planning and the execution of the performed action, but is also recruiting the left dPMC to perform the action successfully. It seems, therefore than when a precision grip is performed with the LNH a ‘bridge’ across hemispheres at the level of the dPMC is activated. In other words, the hemisphere devoted to manage the ongoing action recruits resources also from the other hemisphere. Support to this contention comes from previous neuroimaging evidence suggesting that during the performance of grasping movements with the left hand only the dPMC within the right hemisphere appears to be significantly activated (Begliomini et al., 2008).

These neurophysiological and neuroimaging findings demonstrating the key role of dPMC in controlling distal actions (Raos et al., 2003, 2004) may provide an explanation for these effects and compelling evidence that there are neurons in the distal forelimb representation within area F2 that are specifically selective for the type of prehension required to grasp an object (Raos et al., 2003). They also underline the relevant role dPMC in the on-line control of goal-related hand movements. The increase in connectivity between the dPMC areas outlined by our studies for the ‘left–right’ direction could indicate that they are activated differentially as the non-dominant left-hand is less skilled and requires more control to perform the tasks.

To conclude, our results shed new light on the complex intra- and inter-hemispheric interplay that takes place within the cortical motor system underlying grasping actions. The results not only validate neurophysiological and neuroimaging data at the level of the grasping circuit, but also allows examining the organization of areas for grasping movements performed with either the dominant or the non-dominant hand in both hemispheres. In the future a DCM approach may serve to assess and evaluate similar processes in left-handers as to understand whether the neural organization of grasping may change with respect to handedness.

## Conflict of Interest Statement

The authors declare that the research was conducted in the absence of any commercial or financial relationships that could be construed as a potential conflict of interest.

## Footnotes

1 http://www.fil.ion.ucl.ac.uk/spm

2 http://www.mni.mcgill.ca
