# Supplementary material for: Exploring manual asymmetries during grasping: a dynamic causal modeling approach
Source: Front Psychol. 2015 Feb 24;6:167. doi: 10.3389/fpsyg.2015.00167 (PMC4338815; doi:10.3389/fpsyg.2015.00167)
Supplement: Supplementary file 1 [file table_1.docx]

| Table 1. Subject-specific MNI coordinates acting as a reference point for VOI selection in the DCM analysis. AIP: Anterior IntraParietal; vPMC:ventral PreMotor Cortex; dPMC: dorsal PreMotor Cortex; M1: Primary Motor Cortex | | | | | | | | | | | | | | | | | | | | | | | | |
| --- | --- | --- | --- | --- | --- | --- | --- | --- | --- | --- | --- | --- | --- | --- | --- | --- | --- | --- | --- | --- | --- | --- | --- | --- |
|  | **AIP** | | | | | | **vPMC** | | | | | | **dPMC** | | | | | | **M1** | | | | | |
|  | ***Left*** | | | ***Right*** | | | ***Left*** | | | ***Right*** | | | ***Left*** | | | ***Right*** | | | ***Left*** | | | ***Right*** | | |
| **1** | -38 | -46 | 45 | 38 | -46 | 55 | -52 | 3 | 28 | 61 | 10 | 13 | -19 | -6 | 67 | 31 | -10 | 58 | -25 | -36 | 73 | 37 | -16 | 43 |
| **2** | -35 | -46 | 49 | 47 | -39 | 55 | -55 | 7 | 28 | 57 | 7 | 16 | -19 | -13 | 67 | 24 | -13 | 64 | -38 | -23 | 55 | 37 | -23 | 43 |
| **3** | -42 | -39 | 37 | 37 | -43 | 37 | -61 | 7 | 10 | 51 | 7 | 10 | -12 | 0 | 64 | 28 | -13 | 70 | -35 | -16 | 40 | 41 | -16 | 37 |
| **4** | -48 | -39 | 34 | 34 | -46 | 55 | -52 | 20 | -5 | 54 | 10 | -5 | -15 | 3 | 67 | 18 | -10 | 70 | -38 | -23 | 49 | 41 | -23 | 55 |
| **5** | -48 | -46 | 49 | 41 | -39 | 40 | -48 | 20 | 22 | 51 | 27 | 7 | -29 | -13 | 70 | 34 | -10 | 67 | -45 | -16 | 52 | 37 | -20 | 49 |
| **6** | -48 | -46 | 45 | 47 | -43 | 55 | -52 | 20 | -5 | 61 | 10 | -2 | -29 | -10 | 64 | 31 | -13 | 64 | -38 | -20 | 46 | 47 | -16 | 40 |
| **7** | -48 | -46 | 51 | 37 | -46 | 51 | -58 | 10 | 4 | 51 | 20 | -2 | -29 | -13 | 70 | 34 | 0 | 61 | -45 | -16 | 52 | 44 | -13 | 43 |
| **8** | -38 | -59 | 46 | 37 | -49 | 55 | -48 | 16 | 13 | 54 | 36 | 13 | -22 | -13 | 73 | 31 | -10 | 70 | -35 | -20 | 49 | 34 | -20 | 49 |
| **9** | -38 | -46 | 51 | 38 | -46 | 55 | -58 | 13 | 7 | 61 | 10 | 7 | -12 | -6 | 64 | 18 | -10 | 64 | -35 | -26 | 64 | 37 | -20 | 49 |
| **10** | -35 | -53 | 34 | 38 | -49 | 37 | -48 | 20 | 22 | 51 | 10 | 19 | -15 | -3 | 58 | 18 | -10 | 58 | -38 | -23 | 52 | 31 | -33 | 52 |
| **11** | -32 | -49 | 52 | 38 | -39 | 43 | -48 | 20 | 16 | 54 | 20 | 22 | -19 | -13 | 64 | 31 | -6 | 58 | -38 | -23 | 58 | 31 | -33 | 52 |
| **12** | -45 | -33 | 40 | 44 | -39 | 55 | -48 | 23 | 13 | 41 | 3 | 16 | -21 | -20 | 58 | 38 | -20 | 58 | -45 | -23 | 52 | 34 | -20 | 46 |
| **13** | -38 | -49 | 51 | 41 | -46 | 51 | -58 | 20 | 7 | 54 | 20 | 1 | -29 | -13 | 70 | 14 | 0 | 70 | -45 | -16 | 52 | 37 | -20 | 52 |
| **14** | -42 | -59 | 40 | 41 | -45 | 40 | -48 | 20 | 16 | 61 | 13 | 1 | -22 | 3 | 64 | 21 | 7 | 67 | -38 | -26 | 67 | 28 | -26 | 70 |
| **15** | -35 | -46 | 49 | 37 | -46 | 34 | -58 | 17 | 1 | 47 | 13 | 10 | -29 | -13 | 67 | 31 | -13 | 67 | -38 | -13 | 40 | 34 | -30 | 55 |
| **16** | -51 | -46 | 45 | 57 | -39 | 43 | -48 | 23 | 25 | 51 | 27 | 7 | -25 | -13 | 73 | 34 | -10 | 67 | -45 | -16 | 52 | 41 | -16 | 52 |
